# High precision mass measurements for wine metabolomics

**DOI:** 10.3389/fchem.2014.00102

**Published:** 2014-11-13

**Authors:** Chloé Roullier-Gall, Michael Witting, Régis D. Gougeon, Philippe Schmitt-Kopplin

**Affiliations:** ^1^UMR PAM Université de Bourgogne/AgroSup Dijon, Institut Universitaire de la Vigne et du VinJules Guyot, Dijon, France; ^2^Research Unit Analytical BioGeoChemistry, Department of Environmental Sciences, Helmholtz Zentrum MünchenNeuherberg, Germany; ^3^Chair of Analytical Food Chemistry, Technische Universität MünchenFreising-Weihenstephan, Germany

**Keywords:** wine, non-targeted metabolomics, UPLC-Q-ToF-MS, FTICR-MS, MS/MS, multivariate data analysis

## Abstract

An overview of the critical steps for the non-targeted Ultra-High Performance Liquid Chromatography coupled with Quadrupole Time-of-Flight Mass Spectrometry (UPLC-Q-ToF-MS) analysis of wine chemistry is given, ranging from the study design, data preprocessing and statistical analyses, to markers identification. UPLC-Q-ToF-MS data was enhanced by the alignment of exact mass data from FTICR-MS, and marker peaks were identified using UPLC-Q-ToF-MS^2^. In combination with multivariate statistical tools and the annotation of peaks with metabolites from relevant databases, this analytical process provides a fine description of the chemical complexity of wines, as exemplified in the case of red (Pinot noir) and white (Chardonnay) wines from various geographic origins in Burgundy.

## Introduction

The large amount of wine analytical targeted analyses have definitely contributed to a better knowledge of the wine chemistry (Flamini, 2013). Alternatively, metabolomics approaches have shown great potential for the study of grape or wine (Gougeon et al., 2009). The concept of wine omics (Metabolomics: Wine-omics, 2008), for the untargeted wine analyses coupling NMR and GC-MS were followed by the oenomics approaches, described by Gougeon et al. (2009), exemplified by the revealing of the metabologeography expression of cooperage oak wood by FTICR-MS. The composition of wine is determined by a complex interplay between environmental factors, genetic factors (grape varieties) and viticultural practices (Atanassov et al., 2009; Roullier-Gall et al., 2014a), and metabolomics offers the toolbox for integrated analyses of the wine composition resulting from all of these factors. Holistic analyses require access to sensitive and powerful instruments, therefore metabolomics typically employs high resolution techniques like FTICR-MS, LC-MS, GC-MS, and NMR spectroscopy combined with multivariate statistics. The advantages and disadvantages in terms of selectivity, sensitivity, dynamic range, robustness, and accuracy of these different analytical tools have been well described (Heeren et al., 2004; Hertkorn et al., 2007; Cevallos-Cevallos et al., 2009; Hong, 2011; Theodoridis et al., 2012a).

Vine and wine are well-documented fields of chemistry and various NMR-based or MS-based studies have been published in recent years. As examples, NMR now allows the differentiation of important parameters like the grape variety, geographical origin and year of vintage (Godelmann et al., 2013) whereas FTICR-MS enables the separation of wines according to the geographical origin inside proximate areas (Roullier-Gall et al., 2014a,b).

FTICR-MS is a powerful technique for studying wine, mostly employed in direct infusion experiments to benefit from its ultrahigh resolving power and mass accuracy (Gougeon et al., 2009; Liger-Belair et al., 2009; Roullier-Gall et al., 2014b). However, as a major disadvantage, direct infusion techniques do not allow to discriminate between isomeric and isobaric compounds (Forcisi et al., 2013). If a chromatographic separation is not applied at the first stage, a large number of molecules are subjected simultaneously to the ionization process causing ion suppression for numerous analytes and the inability to separate isobaric and isomeric substances (Forcisi et al., 2013; Gika et al., 2014). Due to this fact, chromatographic separation prior to MS-analysis is particularly important for both targeted and non-targeted metabolomics, and is already used for untargeted metabolomics analysis of grape to differentiate grape berry ripening and post-harvest withering (Toffali et al., 2011), for wine authentication (Rubert et al., 2014), or for the understanding of the terroir impact in wine (Tarr et al., 2013).

LC-MS is designed for the analyses of non-volatile compounds present in complex matrices such as amino acids (Tolin et al., 2012), phenolic compounds (Jaitz et al., 2010), fatty acids (Della Corte et al., 2013). A large number of LC-MS metabolite profiling studies have been realized using a combination of UPLC with time-of-flight mass spectrometry (TOF-MS) (Grata et al., 2008; Fontana et al., 2011; Vaclavik et al., 2011; Forcisi et al., 2013; Rubert et al., 2014). LC-Q-ToF–MS was applied in many wine studies for targeted analyses such as the separation and detection of resveratrol (Wang et al., 2002), of phenolic compounds (Püssa et al., 2006; Sun et al., 2006; Prosen et al., 2007; Gruz et al., 2008; Jaitz et al., 2010) or toxins (Zöllner et al., 2000). In contrast, very few studies have used this technique for untargeted metabolomics analyses of grapes and wines, as shown for instance by Arapitsas et al. or Vaclavik et al. for the understanding of the mechanism of wine micro-oxygenation or to discriminate wines according to grape varieties (Vaclavik et al., 2011; Arapitsas et al., 2012). Such approach combines the highest chromatographic resolution with an excellent sensitivity, fast data acquisition and high mass accuracy (Forcisi et al., 2013; Gika et al., 2014). However, it does not refer to the highest resolution that can only be achieved by FTICR-MS, which typically requires higher time domains and thus do not fully exploit the potential of fast UPLC.

MS-based techniques are in perpetual development, improving the mass resolution, the precision and velocity of acquisition, thus enabling improved detection and identification (Allwood and Goodacre, 2010; Forcisi et al., 2013). The selection of the ionization mode and sorbent materials will have a significant effect on the obtained metabolic profile. Some molecules are ionized more efficiently in one ionization mode. In order to cover the widest range of compounds separated via LC, UPLC-Q-ToF-MS combined with both reversed phase and hydrophilic interaction chromatography represents a powerful analytical platform (Theodoridis et al., 2012a; Forcisi et al., 2013). Reversed-phase (RP) liquid chromatography has been highlighted as the mostly used separation mode for the metabolome analysis (Vaclavik et al., 2011; Theodoridis et al., 2012a,b; Gika et al., 2014). RP separation covers a large part of the metabolome, and at the same time provides the most reliable, robust and sophisticated LC stationary phases, while the number of available chemistries, geometrical characteristics of particles and columns surpasses the corresponding numbers for all other modes. Alternative separation mechanisms are required to separate polar analytes. Hydrophilic interaction chromatography (HILIC) provides separations complementary to those obtained by RPLC–MS in that early eluting analytes in the RP mode are often well retained by HILIC (Forcisi et al., 2013; Müller et al., 2013; Gika et al., 2014).

The current bottleneck in MS based metabolomics is the structural identification of compounds associated with the detected masses. The high levels of resolution and mass accuracy make Q-ToF-MS appropriate for combined MS profiling and MS/MS analysis, thus allowing the complete metabolite discovery process from the untargeted analyses to the structural elucidation of markers (Allwood and Goodacre, 2010; Vaclavik et al., 2011; Rubert et al., 2014). Novel approaches for the analysis of MS/MS spectra are currently developed, e.g., *in silico* fragmentation, offering interesting alternatives for metabolite identification.

The development of non-targeted combined metabolomics approaches have already demonstrated its importance for the metabolite coverage, validation of data and identification of biomarkers, meaning that no method can describe the whole metabolome alone (Theodoridis et al., 2012a; Forcisi et al., 2013; Müller et al., 2013). In this study, we present a complete workflow based on RP-UPLC-Q-ToF-MS and on the exact mass measurement by FTICR-MS, together with multivariate statistics and the usage of *in silico* fragmentation for non-targeted metabolomics analyses of wines. We show that this workflow is at the forefront of wine metabolomics, enabling differentiation of wine from various geographic origins in Burgundy and exemplified here through the identification of common metabolites from wines native to five different producers in Burgundy.

## Materials and methods

### Wines samples

A total of 152 samples of bottled white and red wines from different appellations in Burgundy were analyzed. White wines (Chardonnay) and red wines (Pinot Noir) were sourced from five different producers in Burgundy (Chablis, two different Meursault, Corton Charlemagne and Vosne-Romanée). They cover vintages from 1934 to 2012. All samples were collected under controlled argon atmosphere and stored in 2 ml vials at 6°C prior preparation for analyses (see below).

### FTICR-MS metabolic profiling

High-resolution mass spectra were acquired on a Bruker solariX Ion Cyclotron Resonance Fourier Transform Mass Spectrometer (FTICR-MS) (BrukerDaltonics GmbH, Bremen, Germany) equipped with a 12 Tesla superconducting magnet (Magnex Scientific Inc., Yarnton, GB) and a APOLO II ESI source (BrukerDaltonics GmbH, Bremen, Germany) operated in the negative ionization mode. The negative ion mode fingerprints showed greater variety in the composition and abundance of compounds in the analyzed wines and a smaller number of adducts, as well as higher resolution compared to positive ionization. 20 μL of the samples were diluted in 1 ml of methanol prior to injection and introduced into the microeletrospay source at a flow rate of 120 μL.h^−1^. Spectra were externally calibrated on clusters of arginine (10 mg.L^−1^ in methanol). Further internal calibration was performed for each sample by using ubiquitous fatty acids, reaching mass accuracies lower than 0.1 ppm in routine day-to-day measurement (Gougeon et al., 2009; Roullier-Gall et al., 2014a,b). Spectra were acquired with a time domain of 4 mega words over a mass range of m/z 100 to 1000. 500 scans were accumulated for each sample.

### FTICR-MS pre-processing

The FTICR mass spectra were exported to peak lists with a cut-off signal-to-noise ratio (S/N) of 4. Peak alignment was performed with maximum error thresholds of 1 ppm and filtered for masses occurring in minimum of 10% of all samples. In total, 281432 and 21419 masses composed the final matrix before and after filtration, respectively.

### UPLC-Q-ToF-MS metabolic profiling

1950 μL of the samples were mixed with 50 μL of acetonitrile (ACN) prior to UPLC-Q-ToF-MS analyses. Metabolites were separated using a Waters Acquity UPLC system coupled to a Bruker maXis UHR-ToF-MS. A reversed-phase (RP) separation method was employed. In RP mode, middle to non-polar metabolites were separated using a BEH C8 column (150 mm × 2.1 mm ID). Buffer A consisted of 10% acetonitrile (ACN) in water and buffer B of 100% ACN, both with 0.1% formic acid. Detection was carried out in negative ionization mode with the following parameters: Nebulizer pressure = 2.0 bar, dry gas flow = 8.0 l/min, dry gas temperature = 200°C, capillary voltage = 3500 V, end plate offset = −500 V, mass range = 50–1200 m/z.

### UPLC-Q-ToF-MS data pre-processing

Calibration, alignment and peak picking of individual LC-MS runs were performed using the Genedata Expressionist for MS 8.0 software (Genedata AG, Basel, Switzerland). Internal recalibration was based on 1:4 diluted low concentration tune mix (Agilent, Waldbronn, Germany), which was injected prior to each run using a 6-port valve mounted to the MS. Individual steps of data pre-processing are described in the Results and Discussion Section. Briefly, the complete processing consisted of three stages: Stage 1 performed chemical noise subtraction; Stage 2 performed recalibration and alignment and Stage 3 achieved peak picking and export.

### UPLC-Q-ToF-MS and FTICR-MS alignment

Alignment of both data types was performed using a custom Perl script. For each matching masses between UPLC-Q-ToF-MS and FTICR-MS, the exact mass error was calculated. If the error was smaller than a set threshold (detailed in results and discussion part), the masses were supposed to be the same.

### Statistical analysis

Filtering of masses was performed in MS Excel 2010 (Microsoft, Redmond, USA). All further statistical analyses were performed with Genedata Expressionist for MS 8.0 (Genedata, Basel, Switzerland). Principal Component Analysis (PCA) is an unsupervised method with the capacity to reduce the complexity of a huge dataset. Its goal is to extrapolate important information and display it as a set of new independent variables called principal components. This method discloses the similarity pattern of the observations or variables.

### MS/MS spectra processing

Marker peaks from statistical analyses were subjected to tandem MS. Target lists for fragmentation experiments were converted to MS/MS acquisition methods using MetShot (Neumann et al., 2013). After acquisition, MS/MS spectra were manually extracted using Bruker Data Analysis 4.1 (Bruker Daltonic, Bremen, Germany) and files were exported to.mgf format and converted to MetFusion batch files using a custom Perl script.

## Results and discussion

### Prerequisites for successful metabolic profiling of wines using UPLC-Q-ToF-MS

For unambiguous profiling of the metabolic signature of wines using UPLC-Q-ToF-MS several points have to be taken into account. First, due to the number of samples we aimed to profile, we had to split them into several batches. We decided to consider batches according to appellations, thus leading to four unequally sized batches of 12, 24, 29, and 87 samples. This approach ensured that similar samples are comparable even, if batch-to-batch differences occur. However, to minimize batch-to-batch effects and to have a marker for comparison, we used quality control samples, which are a pool of all samples analyzed (Gika et al., 2014; Naz et al., 2014). These QC samples were injected 10 times prior to each batch in order to equilibrate the system and injected every 10 samples to monitor retention and intensity shifts. Lastly, to make use of the high resolving power of the employed mass spectrometer, prior to each analytical run a calibrant was automatically injected allowing individual recalibration for the correction of mass shifts. All of these points helped to guarantee the highest possible quality of data.

### Pre-processing of FTICR-MS data

**Figure 1:**
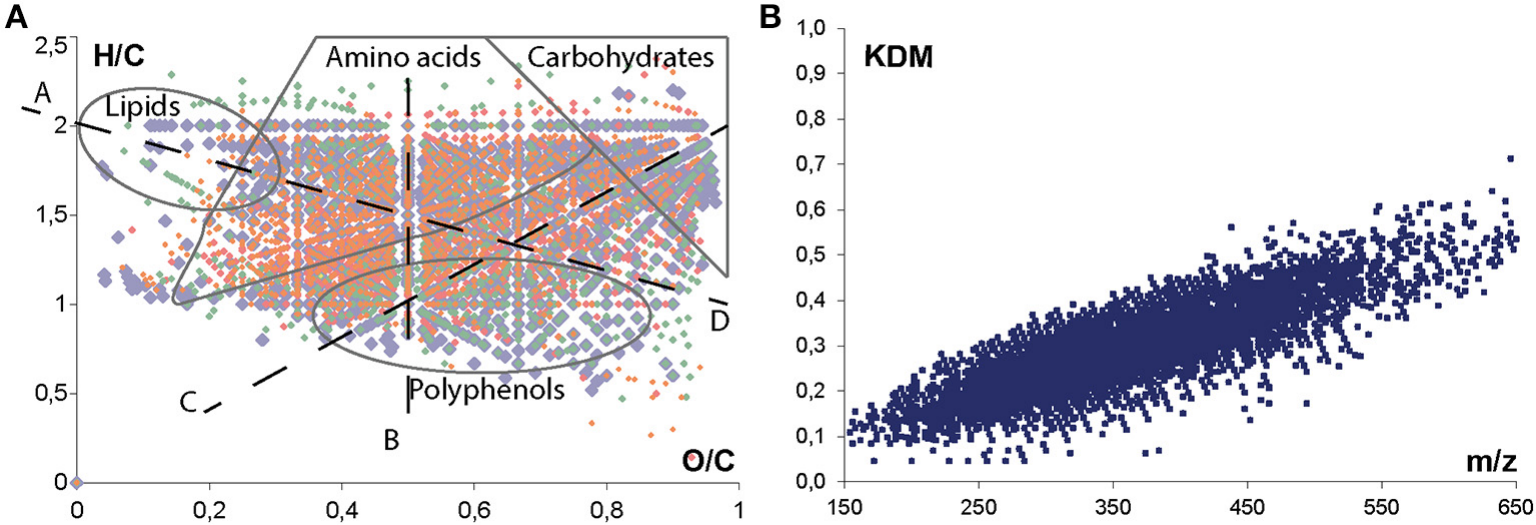
**Van krevelen diagram (A) and Kendrick Mass Defects plot (B) representations of (−) FTICR-MS data corresponding to the complete data set (152 samples) after filtration; annotation of distinctive areas and lines of regions of predominant appearances of metabolite classes and formal chemical alterations: (A) methylation/demethylation (CH_2_); (B) hydrogenation/dehydrogenation (H_2_); (C) hydratation/condensation (H_2_O) and (D) oxidation/reduction (O)**. Colors correspond to elemental formulas composition: (CHO) blue; (CHOS) green; (CHON) red; and (CHONS) orange.

Non-targeted FTICR-MS analysis generates a tremendous amount of data and requires pre-treatment prior to the application of statistical tools. Raw data were first aligned in order to discover occurring patterns, to identify outliers, to reduce the dimensionality of the data, and also to compress large datasets into smaller and more discernable ones (Lucio, 2009). A common approach for the identification of unknown molecules is the calculation of possible elemental formulas. Molecular formulae were calculated using an in-house software tool with the following chemical constraints: N rule; O/C ratio ≤ 1; H/C ratio ≤ 2n + 2; element counts: C ≤ 100, H ≤ 200, O ≤ 80, N ≤ 3, S ≤ 3, and P ≤ 1 (Gougeon et al., 2009; Schmitt-Kopplin et al., 2010; Roullier-Gall et al., 2014a,b). On the total of 21419 masses composing the matrix after filtration, 8455 unambiguous elemental formulas were found. Due to the expected high complexity of the metabolome, visualization strategies dealing with very complex data matrices have been adapted (Hertkorn et al., 2007). The Van Krevelen diagram displays the hydrogen/carbon (H/C) vs. oxygen/carbon (O/C) ratios of these elemental formulas and provide a commonly used qualitative description of the molecular complexity of wine data (Gougeon et al., 2011; Roullier-Gall et al., 2014a,b). This plot enables the localization of areas covering metabolite classes, which are specified by different elemental compositions, enabling a representation of a sample's composition (Figure 1A). The richness of the observed mass gives evidence of the compositional diversity of molecules as carbohydrates, polyphenols or amino acids and chemical alteration as hydrogenation / dehydrogenation (Figure 1A ligne B) for example. A second plot for visualization and interpretation of ultrahigh resolution mass spectrometry data is the CH_2_ Kendrick plot (Hertkorn et al., 2007), which is based on distinct mass defects calculated from each elemental composition (Figure 1B). Molecules of different elemental composition differ in their mass defect and it became possible distinguish homologous series of compounds from each other.

### Automated data pre-processing of UPLC-Q-ToF-MS data

A total of 286 chromatograms were obtained, including blank injections and QC samples. To analyze this amount of data, automated pre-processing is needed. Several open-source software packages exist for this task. We employed Genedata Expressionist for MS 8.0, which allows workflow-based data processing in a user-friendly graphical user interface. Our processing strategy consisted in three stages. Stage 1 mainly aims at data reduction by use of chemical noise subtraction. This is performed in a so-called repetition activity, which handles only one chromatogram at a time, enabling faster processing, because memory of the employed computer system is optimally used and no files have to be paged to cache plates. Additionally, this prevents an overhead of the memory of very big studies with >250 chromatograms. The noise subtracted data is stored in the proprietary.sbf format yielding a 124 fold data reduction compared to initial raw data. These noise subtracted chromatograms served as input for the second stage. All files were internally recalibrated on the calibrant segment in the retention time range from 0.05 to 0.3 min. A mass error <5 ppm could be achieved for the calibrant masses. All of the chromatograms were then aligned in the RT direction. A pairwise alignment tree was constructed to guide the alignment, with the following procedure: two most similar chromatograms are first aligned, followed by the next more similar and so on until all chromatograms have been aligned. This methodology is especially useful for samples with high chemical diversity, which is the case for wine. Intermediate results were again stored in the.sbf format. In the third and last stage, noise subtracted, recalibrated and aligned spectra underwent peak picking. All of the detected features were combined into isotopic clusters and data were finally exported to.gda and.xlsx files for statistical analyses and alternative analyses, respectively.

### Metabolite annotation using different metabolomics databases

**Figure 2:**
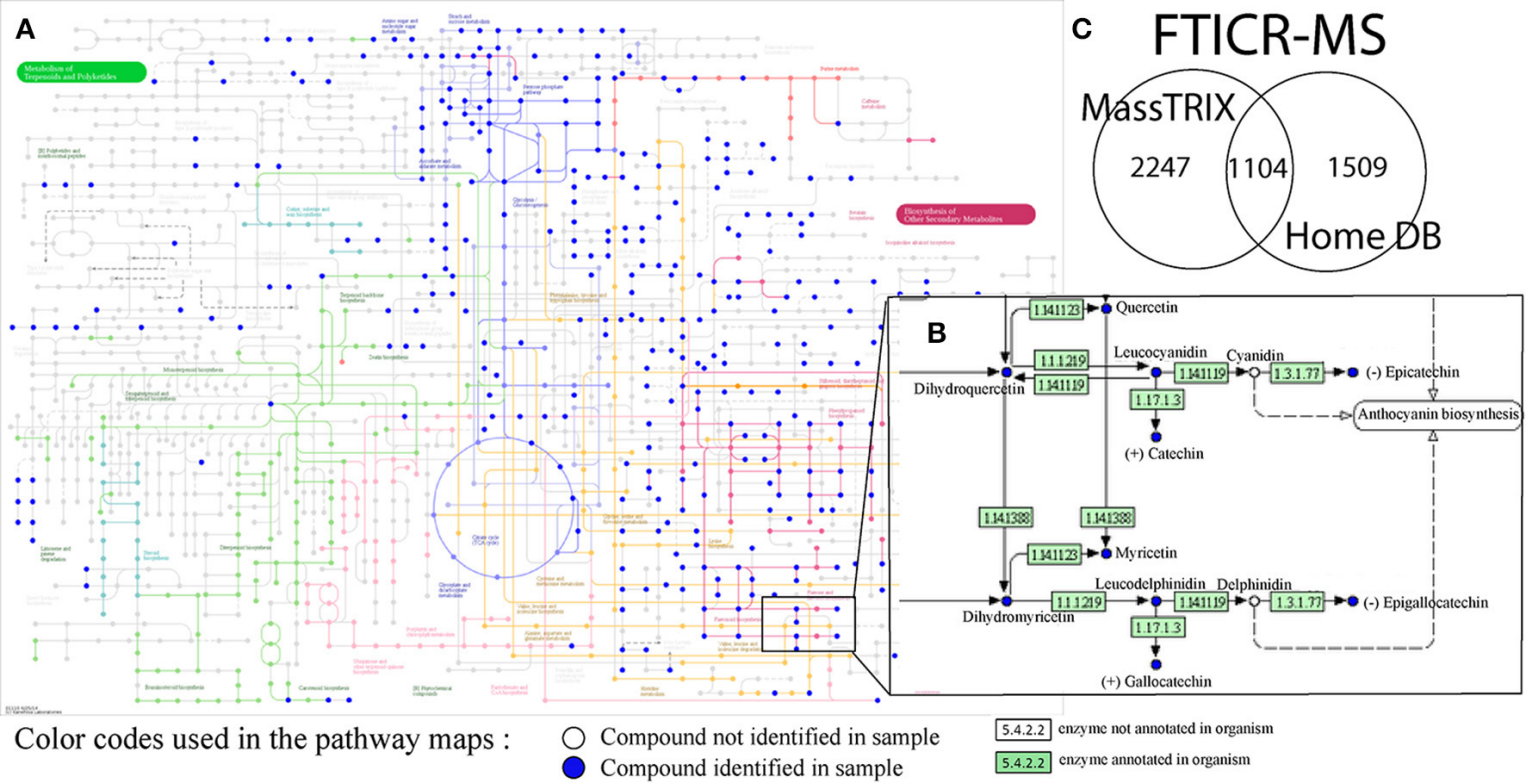
**(A)** Pathways for the biosynthesis of secondary metabolites from KEGG for the *vitis vinifera* organism and **(B)** enlargement of a portion of flavonoid biosynthesis pathways, with annotated compounds (blue dots) possibly corresponding to detected masses from all of the (−) FTICR-mass spectra; **(C)** Venn diagrams showing the convergence between annotations from different data bases (MassTRIX vs. home build wine data base, top).

A first overview of potential structures corresponding to detected metabolites was obtained by metabolite annotation using both the MassTRIX interface and a home-build (including grape and wine) metabolite database (Suhre and Schmitt-Kopplin, 2008; Wägele et al., 2012; Roullier-Gall et al., 2014a,b) (Figure 2). A search against KEGG, HMDB and LipidMaps with maximum error of 3 ppm was performed. MassTRIX and KEGG enable the visualization of compounds annotation on pathways of a chosen organism (*Vitis vinifera* in this example) (Figures 2A,B). In total, 3351 detected masses from FTICR-MS could be annotated using MassTRIX, whereas 2613 detected masses could be annotated using our home-build metabolite database (Figure 2C). Around 22% of total detected features from FTICR-MS are detected by both MassTRIX and homemade data base. Only few features, 119, from UPLC-Q-ToF-MS were annotated using MassTRIX, comparing to the 3351 masses from FTICR-MS (Figure 2C) proving the necessity of the alignment of both datasets.

### UPLC-Q-ToF-MS and FTICR-MS alignment

**Figure 3:**
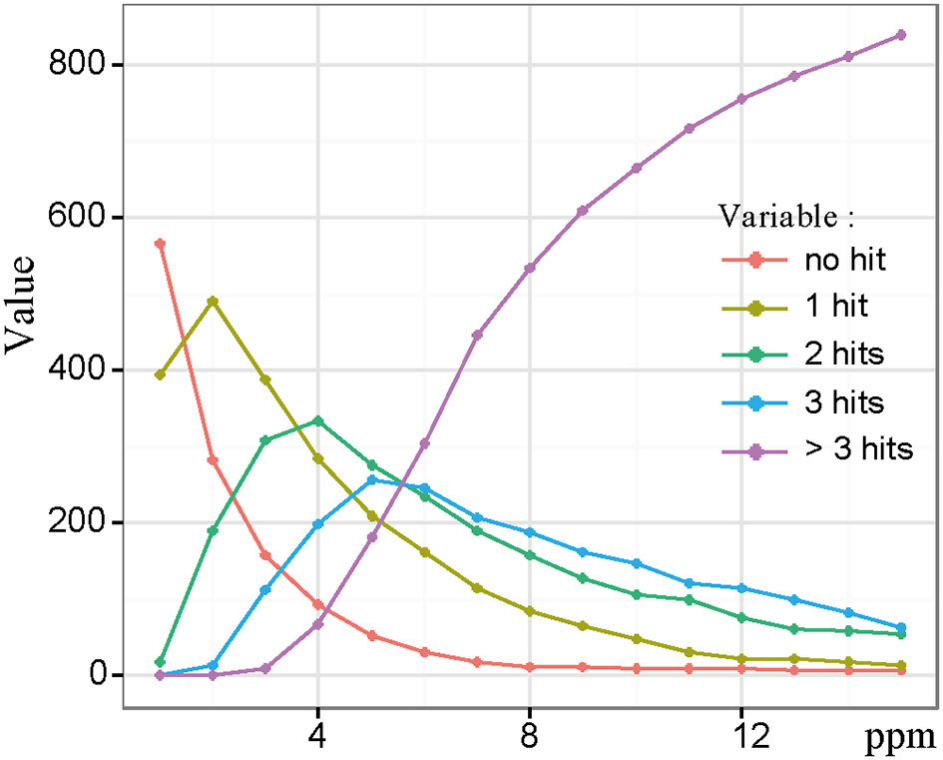
**Statistics of LC-FT-MS alignment showing number of hits between LC-MS and FTICR-MS masses as function of error**. With higher errors number of clusters with no hits decreased, but also the number of clusters with multiple hits increased. We have chosen an error of 3 ppm, which is the last point were unique hits exceed multiple hits.

Classical approaches where FT-MS would have been coupled to LC, would have required a reduction of the resolution in order to increase the scan rate. Here, by doing the UPLC-UHR-ToF-MS and FTICR-MS combination *in silico*, we get the best of both worlds, i.e., a high resolution in LC and ultra-high resolution provided by FTICR-MS. In order to compare both datasets and confirm exact masses for the UPLC-Q-ToF-MS data, we performed the alignment of both datasets using a custom Perl script. In order to find optimal alignment conditions we used maximum error thresholds from 1 to 15 ppm in 1 ppm steps and compared the number of unique, double, triple, or more hits. The procedure of alignment gives us the remarkable advantage of combining exact mass information from FTICR-MS with retention time data from UPLC-UHR-ToF-MS. With this information, elemental formulas related to unknown metabolites can also be supplied with putative chemical structures that are not necessarily covered by metabolites present in the databases used for annotation. Figure 3 shows a plot of the number of UPLC-Q-ToF-MS features with an exact mass hit in the ICR-FT/MS data set. A feature could either have no hit, one, two, three, or more hits, which are depicted individually. 2 ppm had the highest number of features with a unique hit. However, we used 3 ppm for further investigation, because it is the last error having higher number of unique hits compared to features with more hits. This enabled us to cover as many different solutions as possible, without having too many false positives which were observed at the higher range of the thresholds (Figure 3). Surprisingly, we still had 158 LC-MS features with no FTICR-MS hit. These were further investigated regarding their mass and intensity. A possible explanation is that we used different mass ranges for the two different methods. However, all of these LC-MS features were in the mass range of the FTICR-MS, from m/z 124.9920 to 688.9692. Since no specific trends in either intensities or retention time regions could be identified (data not shown), we therefore concluded that these features were molecules likely easily suppressed in direction infusion ESI, thus showing the added value of a chromatographic separation, and more generally the complementarity between the two mass spectrometry methods.

### Statistical analysis of FTICR-MS and UPLC-Q-ToF-MS data

**Figure 4:**
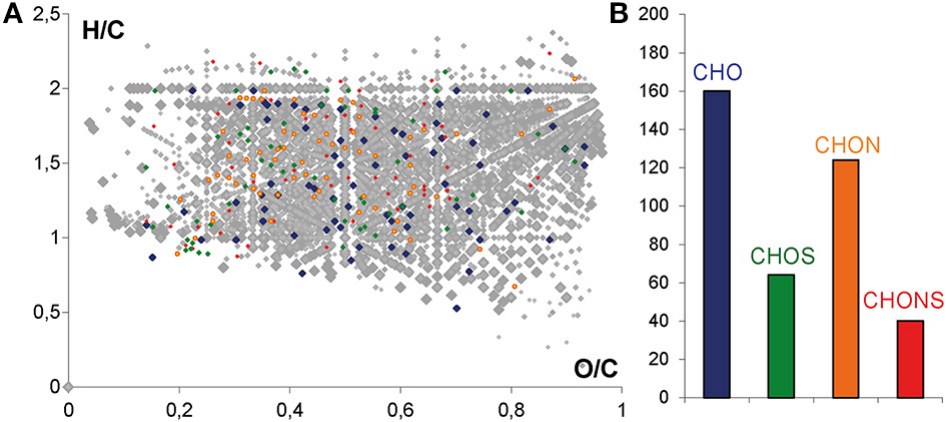
**(A)** H/C vs. O/C van Krevelen diagram of the masses from FTICR-MS (gray) and common masses from UPLC-QToF-MS and FTICR-MS. **(B)** Histograms of the relative frequency of common masses from UPLC-QToF-MS and FTICR-MS. Color code: CHO, blue; CHOS, green; CHON, red; CHONS, orange.

Mass lists obtained from all of the samples were merged into one data matrix and the calculated molecular compositions of all m/z features detected in UPLC-Q-ToF-MS were visualized in a van Krevelen diagram (in color) after alignment with the FTICR-MS mass list (in gray) (Figure 4A). The molecular composition of all m/z features detected in (-) FTICR-MS appears very complex (gray), with regions of carbohydrates, amino acids and polyphenolics being extremely occupied (Figures 1A, 4A). A convergence between mass spectrometry data sets can be observed even if the number of m/z features detected in UPLC-Q-ToF-MS (in color) is significantly lower than m/z features from FTICR-MS (in gray) (Figure 4A), the peak abundances and the distributions for CHO, CHOS, CHON, and CHONS were characteristic of wine sample (Figure 4B) with a majority of CHO compounds, follow by CHON compounds (Gougeon et al., 2009; Roullier-Gall et al., 2014b).

**Figure 5:**
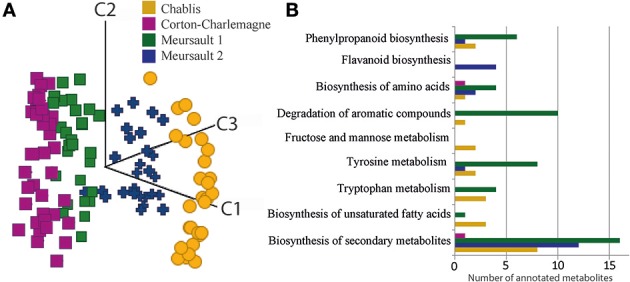
**(A)** PCA score plot for the first three components from RP-UPLC-QToF-MS data sets of white wines, from three distinct appellations in Burgundy, showing the discrimination between wines according to the appellation, regardless of the age of the wine. **(B)** Number of metabolite structures that could be associated with elemental formulas specific to the different geographical origins, in some of the *Vitis vinifera* metabolic pathways, using MassTRIX.

To evaluate the stability of the UPLC-Q-ToF-MS system we used QC samples and checked the RSD of peak intensity of detected peaks in all QC samples. However, we did not use this as filtering criteria because the QC samples consisted of a pool of >120 different wines from different appellations, vintages and ages and therefore the chance was high to dilute out significant and/or discriminant peaks specific to certain samples. Furthermore, samples were measured in two attempts within a timeframe of 1 year. To make both measurements comparable, we used the aligned matrix filtered for masses occurring in minimum 10% of all samples of one measurement run. In total, we obtained 977 features, among which only 692, occurring in at least 50% of all samples, were kept for further statistical analyses. However, differences in intensities between the QC samples from the two measurements were found. In order to take them into account by normalization, we used Z-transformation, which performs unit variance scaling and back transformation. This transformation strongly reduced variation across QC samples from the two measurement regimes, and thus drastically reduced, but not completely removed differences based on different times of measurement. It must be noted that the design of batches was done in a way that it resulted in sample sets that could be analyzed individually, allowing overall comparison of markers. A PCA analysis of wines was performed on RP-UPLC-Q-ToF-MS data for the four series of Burgundy Chardonnay wines, which separated them according to the appellation (Chablis, two different Meursaults and Corton Charlemagne) (Figure 5A). Although this was not the purpose of that paper, this result illustrates the potentiality of non-targeted MS-based analyses for the discrimination of wines according to their appellation of origin (geographical origin of grapes), regardless of the vintage, and thus nicely confirms that wine's metabolic diversity holds various chemical fingerprints that advanced non-targeted metabolomics can read. Possible structural assignments for specific masses for geographical origins (Figure 5B) could be obtained from databases or interfaces such as SciFinder Scholar or MassTrix (Wägele et al., 2012). However, on average more than 80% of the discriminant masses could not be annotated. Through the MassTRIX interface, 56 elemental formulas specific to Chablis wines, 59, 50, and 3 elementals formulas specific to Meursault 1, Meursault 2 and Corton Charlemagne wines, respectively (out of 304, 327, 204, and 267 masses, specific to Chablis, Meursault 1, Meursault 2, and Corton Charlemagne wines, respectively) could be correlated to hypothetical metabolite structures from the various metabolic pathways of the *Vitis vinifera* organism (Figure 5B) (Suhre and Schmitt-Kopplin, 2008; Wägele et al., 2012). If Chablis wines were clearly characterized by hits in each represented pathways (except for the flavanoid biosynthesis), Meursault1 wines were characterized by hits in degradation of aromatic compounds pathways and Meursault2 in Flavanoid biosynthesis pathways. All of the metabolic pathways appeared to be involved in this discrimination and covering a large number of molecular families including polyphenols, fatty acids, carbohydrates, and amino acids (Figure 5B).

### Analysis of marker peaks

Putative annotations of significantly different metabolites only give small evidence on their real structure, because several possible isomers and isobars might exist, especially for secondary metabolites. Therefore, we performed MS/MS experiments on statistically identified markers from the different data sets. We used the R package MetShot to sort given RT and m/z into different subgroups for MS/MS to have a minimum number of acquisition methods that cover all features (Neumann et al., 2013). MS/MS spectra were manually extracted and exported to.mgf files from which MetFusion batch files were generated using a custom Perl script. Identification of metabolites is the current bottleneck of metabolomics (Evans et al., 2009). Although tandem MS libraries are growing fast, numerous metabolites are still missing. The most promising solution to overcome this problem is *in silico* fragmentation. Several tools exist and we used MetFusion for analysis (Tohge and Fernie, 2009; Heinonen et al., 2012; Gerlich and Neumann, 2013; Allen et al., 2014). This web-based tool uses the *in silico* fragmentation tool MetFrag in combination with different MS/MS databases, like MassBank or Metlin. Results were ranked based on spectral similarities to the *in silico* spectrum of compound matching the precursor mass and spectral similarities to spectra stored in different databases. All spectra were uploaded to a MetFusion batch client. To prove the applicability of this approach for metabolite identification we have chosen an example, which will be discussed in more detail.

**Figure 6:**
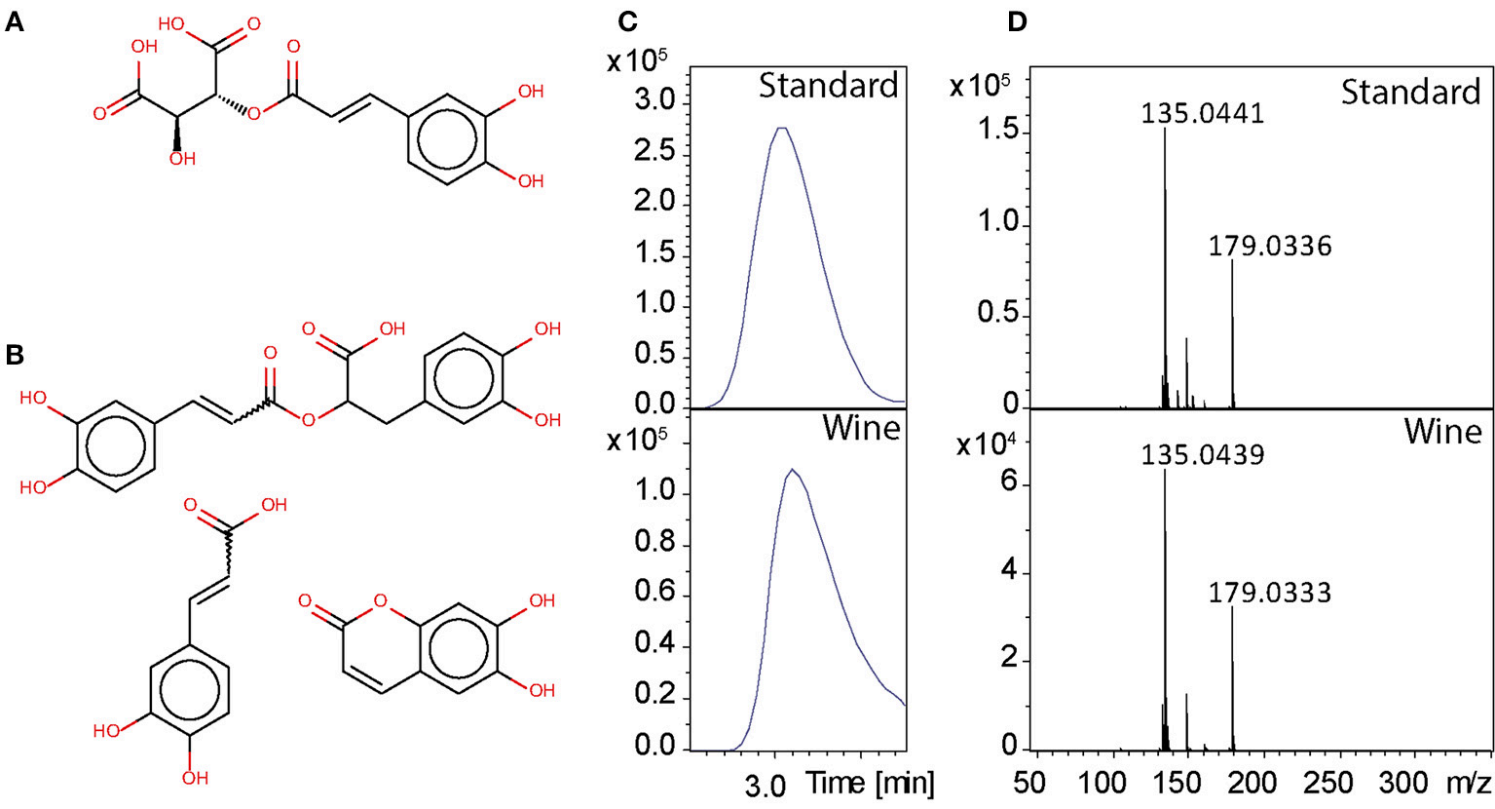
**(A)** Caftaric acid, **(B)** structures with spectral similarities: rosmarinic acid, caffeic acid, and aesculetin. **(C)** Chromatographic peaks, **(D)** MS/MS spectra with data from the standard and from the wine.

As example we show results for m/z 311.0406 at retention time 3.46 min. This mass was putatively annotated as caftaric acid (Figure 6A) with an error of −0.79 ppm. Cafltaric acid is known to take part of the color of white wine and is considered to be one of the strongest natural antioxydant of Chardonnay (Cheynier et al., 2006), and it's concentration is known to decease upon oxidation (Fernández de Simón et al., 2014; Gawel et al., 2014). From FTICR-MS, the exact mass of 311.0408523 was aligned to this feature. Using our MetShot based approach we obtained a MS/MS spectrum suitable for further analysis. We uploaded the most intense fragment peaks to MetFusion, then searched the ChemSpider database with an error of 1 ppm and used the European mirror of MassBank for MS/MS search. Six potential candidates were found and caftaric acid yielded the highest score with 5 explained peaks (Figures 6C,D). Comparison to known MS/MS spectra showed spectral similarities with caffeic acid, rosmarinic acid and aesculetin (Figure 6B). Since we could not find a MS/MS spectrum in other databases for confirmation, we ran the LC-MS analysis of a standard caftaric acid under identical conditions. The comparison of MS/MS spectrum from standard with data from wine, allow to confirm the identity of this peak as caftaric acid.

## Conclusion

The use of Ultra-high performance liquid chromatography coupled with quadrupole time-of-flight mass spectrometry (UPLC-Q-ToF-MS) and Ion Cyclotron Resonance Fourier Transform Mass Spectrometer (FTICR-MS) for non-targeted metabolic profiling and metabolite identification of wine is shown here. Alignment between FTICR-MS and UPLC-Q-ToF-MS was used to obtain retention time informations together with exact mass measurements. The combination of these two methods of metabolic profiling enable a superior resolution and mass accuracy, it provide good groups separation and revealed possible markers for each groups. This approach added the possibility to obtain exact mass for formula calculation with retention time information of unknown molecules, which can add hints about possible molecule structure. Wine is considered as a complex biological system impacted by many environmental conditions related to grape growth, winemaking practices, or to wine storage. From oenological point of view, the use of these two different methods of metabolic pofiling allows the authentication of wine, regardless of the vintage, depending on its geographical origin. These results provide insight novelty in terms of the wine identification by the specific chemical composition of wine from different appellations in Burgundy.

### Conflict of interest statement

The authors declare that the research was conducted in the absence of any commercial or financial relationships that could be construed as a potential conflict of interest.
